# First report on the gall wasp Ophelimus near migdanorum (Hymenoptera, Eulophidae) and its parasitoid *Closterocerus chamaeleon* (Hymenoptera, Eulophidae) in *Eucalyptus globulus* in Bogotá, Colombia

**DOI:** 10.3897/zookeys.902.39213

**Published:** 2020-01-13

**Authors:** Olga Pinzón-Florián

**Affiliations:** 1 Universidad Distrital “Francisco José de Caldas”. Cra 5E 15-82 Bogotá, Colombia Universidad Distrital “Francisco José de Caldas” Bogotá Colombia

**Keywords:** Biological control, introduced pest, natural enemy

## Abstract

Ophelimus near migdanorum, a foliage gall wasp-inducer of *Eucalyptus*, is recorded for the first time in Colombia, infesting both mature ornamental trees of *Eucalyptus globulus* (Labill.) and monoculture plantations in the periurban forest in the city of Bogotá. The parasitoid *Closterocerus chamaeleon* was also emerged from the galled foliage. The spread of this pest and its parasitoid in other *Eucalyptus* species planted in Colombia has not been evaluated.

## Introduction

*Eucalyptus* (Myrtaceae) is the most planted tree genus for commercial purposes in Colombia. *Eucalyptus globulus* (Labill.) was introduced in Colombia at the beginning of the 20^th^ century, and during the decades of 20´s and 30´s was used in reforestation programs to protect soils around the rivers San Cristóbal, San Francisco, and Arzobispo, around the city of Bogotá (Bustos and Venegas 1975). Currently, *E. globulus* is one of the predominant species in the oriental periurban areas of the city, where occupies approximately 2300 has (Secretaria Medio Ambiente – JBB 2007).

*Eucalyptus globulus* in Colombia, up to the present time, records two exotic sap-sucker pest species (Hemiptera: Aphalaridae) attacking the foliage: *Ctenarytaina eucalypti* Maskell (Pinzón et al. 2002) and *Ctenarytaina spatulata* Taylor (Rendón-Mera et al. 2017). To date, no gall-forming insects had been reported in *Eucalyptus* trees planted in Colombia.

## Materials and methods

In order to characterize the damage and associated insects, samples of mature leaves of *E. globulus*, fallen on the ground, were collected during July 2019 in a city park of Bogotá under a number of trees older than 30 years. Also, apical sections 20 cm long, containing young leaves, were collected from mature branches of about ten years old-trees, in a periurban area of the city. Affected trees are growing at 2650 m of altitude and temperatures ranging from 8 to 18 degree Celsius.

The collected samples were maintained in transparent airtight containers, in the Forest Health Laboratory of the Universidad Distrital “Francisco José de Caldas”, at environment temperature ranging from 8 to 18 °C, until the wasps’ emergence.

Emerged wasps were preserved in 95 % ethanol, and samples of the wings and the antennae were prepared for microscopic analysis using PVA mounting media (Bioquip, Rancho Dominguez, California, USA). Voucher specimens are maintained in the Forest Entomological Collection hosted in the Forest Health Lab. under the code CEFUDFJC-LS2019100. Photographs were taken using a 506 Axiocam camera mounted in a Carl Zeiss Discovery 8 Stereo-microscope and stacked using Zen software (version1.04). Size and density of galls in the leaf blade were estimated using the measure option available in the Zen software. A total of 100 fully developed galls were measured and ten leaves were used to estimate average number of galls per square centimeter.

Taxonomic identification of the wasps was performed following characters described in Protasov et al. (2007a, b), the key presented by Borowiec et al. (2019), and the description of Molina-Mercader et al. (2019a, b).

## Results

**Figure 1.**
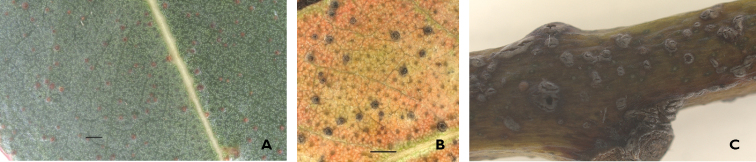
Different stages of gall development on *E. globulus* foliage. **A** Initial stage **B** fully developed galls **C** fully developed galls in the petiole. Scale bar: 1 mm.

Affected leaves display numerous tiny, slightly ellipsoid, galls (44 galls per cm^2;^ average area of a single fully developed gall: 0.06 mm^2^) (Fig. 1). The galls are perceptible to the touch on both the upper and the underside of the leaves and are randomly distributed mostly in the leaf blade but sometimes near the central nerve or in the petiole. Most of the galls are individual, but frequently form clusters of three or four. Likely, the formation of the galls induces premature fall of the foliage under the affected trees. The accumulation of the leaves under the infested trees facilitated the detection, since the height of the trees prevents the direct observation of the foliage. Both a gall-forming species of *Ophelimus* (Eulophidae: Opheliminae) and its parasitoid *Closterocerus chamaeleon* (Girault) (Eulophidae: Entedoninae) were obtained from the collected samples.

**Figure 2.**
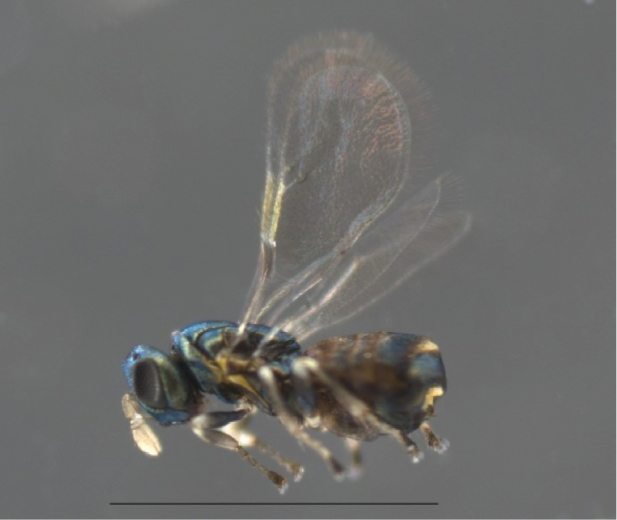
*Ophelimus* sp. Body in lateral view. Scale bar: 1 mm.

Body length of the *Ophelimus* reported here (Fig. 2) range from 1 to 1.1 mm, body color dark brown with metallic shine, and both eyes and ocelli are dark. Legs are dark brown except for the apical region of the femur and tibia and the three first tarsomeres of the meso and metathoracic legs, which are pale. Fore wings have one or two setae on the submarginal vein. Antennal scape is a dark color with flagellomeres and clava being pale brown.

*Ophelimus* reported here does not fit precisely the morphological characters or types of galls reported for *O. maskelli* (Ashmead), *Ophelimus eucalypti* (Gahan) or *O. mediterraneus* Borowiec & Burks. According with the keys compiled by Borowiec et al. (2019), *O. eucalypti* have more submarginal setae on the forewings and is larger than *Ophelimus* sp. On the other hand, although *O. maskelli* induces galls that are visible on both sides of the leaves, they are larger and, also, the wasps have only one submarginal seta on the fore wing. Similarly, *O. mediterraneus* differs in having 2–4 submarginal seta on the fore wings, and although induces small galls, they are only visible on the upper side of the leaves.

Conversely, length of the wasp of *Ophelimus migdanorum* (Molina-Mercader et al. 2019b), general color pattern of the body, number of submarginal seta on the fore wings, as well as size of the galls (small), developing in both sides of the leaves and the petiole, resemble the case reported here. *Ophelimus migdanorum* also attacks *E. globulus*, and considering that Chile is relatively close to Colombia, it may likely be the same species but further detailed comparisons are needed.

**Figure 3.**
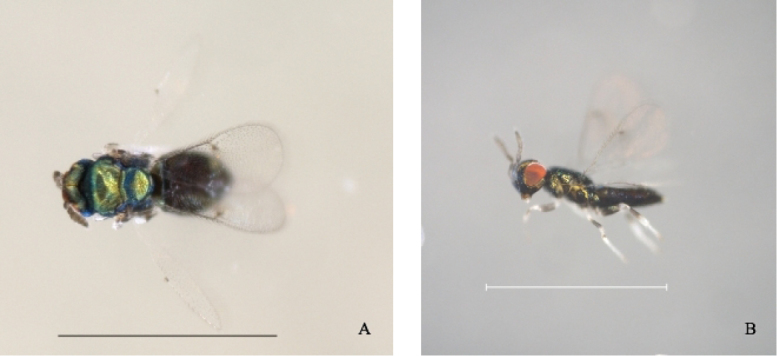
*Closterocerus chamaeleon* emerged from mature *E. globulus* leaves infested by *Ophelimus* sp. **A** Dorsal view **B** lateral view. Scale bars: 1 mm.

A second wasp, found in the same samples of *E. globulus* foliage, was identified as *Closterocerus chamaeleon* (Fig. 3), which, in addition to the keys, was identified using characters included by Molina- Mercader et al. (2019a), such as eye color and body shape, that were useful to fully separate the gall wasp identity from that of the parasitoid.

## Discussion

The genus *Ophelimus* Haliday, native of Australia, comprises approximately 51 species (Noyes 2019) of gall-inducing wasps. To date, four *Ophelimus* species are known to occur outside Australia attacking eucalypts. *Ophelimus eucalypti* was detected in New Zealand attacking *E. globulus* (Withers et al. 2000); *O. maskelli*, is widely distributed in North America and the Mediterranean basin, attacking *E. camaldulensis* and *E. tereticornis* (Burks et al. 2015a, b; Protasov et al. 2007b); *O. mediterraneus* occurring in France, Italy and Portugal attacking *E. globulus*, *E. cinerea*, *E. gunni* and *E. parvula* (Borowiec et al. 2019) and *O. migdanorum* attacking *E. globulus* in Chile (Molina-Mercader et al. 2019). The *Ophelimus* reported here, corresponds to a third of the fortuitous introductions of *Ophelimus* species and its parasitoid in South America, being the previous registers in Argentina (*O. maskelli*, attackin*g E. camaldulensis*, Aquino et al. 2015) and Chile (*O. migdanorum*, attacking *E. globulus*, Molina-Mercader et al. 2019a). Most likely O. nr migdanorum infestation found in Bogotá, along with its parasitoid, correspond to an accidental introduction occurring several years ago but that had gone unnoticed. The size of the galls is small, the wasps are microscopic, and this may have contributed to the fact that the case has gone unnoticed.

*Closterocerus*, on the other hand, is also an Australian genus that comprises 74 species (Noyes 2019), of which *Closterocerus chamaeleon* have been described as an ectoparasite with a narrow range of host species among eulophids forming galls on eucalypts (Protasov et al. 2007a). This parasitoid was effectively introduced from Australia to Israel and Italy, to content *O. maskelli* attacks (Protasov et al. 2007a). However, it was naturally dispersed and reduced *O. maskelli* populations in neighboring countries such as Tunisia and Portugal (Branco et al. 2014), and from there likely to North and South America (Aquino et al. 2015, Molina et al. 2019). In the areas of Bogotá where O. nr migdanorum is being reported, the parasitoid *C. chamaeleon* seems to be well established, as also indicated for *O. maskelli* in California (Burks et al. 2015a). Perhaps, the high effectiveness of *C. chamaeleon* has prevented the intensity of the attacks of *Ophelimus* sp. in urban trees of *E. globulus* in Bogota and surrounding areas, since heavily defoliated areas have not been observed.

Several species of *Eucalyptus*, other than *E. globulus*, are planted in Colombia for industrial purposes; therefore, a further study aimed to detect and estimate the distribution and prevalence of *Ophelimus* sp. and parasitism success of *C. chamaeleon* on those species is valuable.
